# Supplementary material for: Dietary Protein Patterns during Pregnancy Are Associated with Risk of Gestational Diabetes Mellitus in Chinese Pregnant Women
Source: Nutrients. 2022 Apr 13;14(8):1623. doi: 10.3390/nu14081623 (PMC9026337; doi:10.3390/nu14081623)
Supplement: Supplementary file 1 [file nutrients-14-01623-s001.zip › nutrients-1666082-supplementary.pdf]

Dietary protein patterns during pregnancy are associated with risk of gestational diabetes mellitus in Chinese pregnant women

Supplementary materials

Supplementary Table S1 Characteristics of pregnant women by dietary protein patterns[1].

Characteristics	Plant-dairy-eggs (*N*=302)	White meat (*N* =297)	Red meat (*N* =415)	*P*
GDM, *n* (%)				0.332
Yes	49 (16.23)	62 (20.88)	80 (19.28)	
No	253 (83.77)	235 (79.12)	335 (80.72)	
Age, *y*	30.38 ± 5.20	29.75 ± 4.70	30.03 ± 4.68	0.288
< 35, *y*	237 (78.74)	262 (88.22)	352 (84.82)	**0.006**
≥35, *y*	64 (21.26)	35 (11.78)	63 (15.18)	
Gestational age, *week*	25.12 ± 2.23	25.64 ± 2.41	25.55 ± 2.30	**0.016**
Pre-pregnancy BMI, kg/m^2	21.03 ± 3.05	20.42 ± 2.83	20.36 ± 2.82	**0.007**
Overweight or obese, *n* (%)	44 (15.60)	34 (12.19)	41 (10.49)	0.138
Underweight or normal, *n* (%)	238 (84.40)	245 (87.81)	350 (89.51)	
Smoking, *yes*, *n* (%)	9 (3.00)	19 (6.40)	16 (3.89)	0.106
Alcoholuse, *yes*, *n* (%)	14 (4.67)	8 (2.69)	13 (3.16)	0.379
Physical activity,*METs·h/w*	33.19 ± 27.52	31.11 ± 28.46	31.09 ± 25.53	0.540
Family history of diabetes, *yes*, *n* (%)	45 (14.95)	44 (14.81)	61 (14.91)	0.999
**History of GDM, *n* (%)**				0.778
Yes	9 (3.04)	11 (3.72)	9 (2.20)	
No	176 (59.46)	168 (56.76)	241 (58.92)	
Nulliparous	111 (37.50)	117 (39.53)	159 (38.88)	
**Educational level,*n* (%)**				0.403
Senior high school and below	55 (18.84)	56 (19.24)	72 (17.69)	
High or technical secondary school	74 (25.34)	54 (18.56)	85 (20.88)	
Junior college and college	143 (48.97)	166 (57.04)	225 (55.28)	
Postgraduate and above	20 (6.85)	15 (5.15)	45 (6.14)	
**Monthly household income,*n* (%)**				0.442
≤4000 RMB	68 (23.21)	56 (19.38)	85 (21.25)	
4001-6000 RMB	60 (20.48)	69 (23.88)	107 (26.75)	
6001-10000 RMB	69 (23.55)	76 (26.30)	98 (24.50)	
> 10000 RMB	96 (32.76)	88 (30.45)	110 (27.50)	

[1] Values were presented as mean± standard deviation, or proportions.

Supplemental Table S2 Average percentage of total protein intake from individual food group across protein food cluster analysis of 1024 pregnant women from the GDM study[1]

Food group	Plant protein	White meat protein	Red meat protein	*F*	*P*
N (GDM),	302 (49)	297 (62)	415 (80)	1.10	0.332
Grain,%	25.61 ± 9.20	20.55 ± 7.49	25.20 ± 9.31	31.84	**<.0001**
Beans,%	7.17 ± 5.70	5.44 ± 4.09	5.19 ± 4.12	17.61	**<.0001**
Vegetables,%	8.19 ± 3.87	6.33 ± 2.55	6.03 ± 2.81	46.91	**<.0001**
Fruits,%	3.62 ± 2.04	2.44 ± 1.39	2.13 ± 1.23	84.44	**<.0001**
Red meat,%	13.90 ± 6.95	18.66 ± 7.83	31.50 ± 9.86	418.25	**<.0001**
Poultry,%	3.74 ± 2.95	11.18 ± 5.74	4.75 ± 2.94	318.47	**<.0001**
Aquatic products,%	7.29 ± 5.19	14.56 ± 9.17	6.90 ± 4.45	145.19	**<.0001**
Egg,%	9.97 ± 6.15	5.62 ± 3.63	6.48 ± 4.22	73.01	**<.0001**
Dairy,%	13.41 ± 8.16	10.48 ± 6.62	8.63 ± 6.14	41.53	**<.0001**
Nuts and Seeds,%	6.65 ± 6.75	4.28 ± 5.18	2.79 ± 3.49	49.43	**<.0001**

[1]A K-means cluster analysis was used to classify participants into mutually exclusive groups; Naming of clusters was determined by the value which represent the highest consumption of one or two food groups compared with other clusters; Percentage of total protein intake across the each food group was used; Mean±SE.

Supplementary Table S3 Daily nutrients consumption of pregnant women by dietary protein patterns[1].

**Nutrients**[2]	Plant-dairy-eggs (*N*=302)	White meat (*N* =297)	Red meat (*N* =415)	*P*
Total energy, *kcal/d*	1740.76 ± 465.92	1841.58 ± 494.08	1817.10 ± 515.73	**0.032**
Saturated fatty acids, *g/d*	18.80 ± 3.62	20.26 ± 3.67	20.50 ± 4.37	**<.0001**
Monounsaturated fatty acids, *g/d*	26.47 ± 5.36	27.81 ± 5.23	28.71 ± 5.92	**<.0001**
Polyunsaturated fatty acids, *g/d*	21.79 ± 6.11	21.34 ± 5.83	20.07 ± 5.29	**0.0002**
Cholesterol, *mg/d*	400.72 ± 169.93	415.31 ± 153.19	398.36 ± 160.47	0.351
Fiber, *g/d*	12.84 ± 2.97	11.12 ± 2.79	9.82 ± 2.75	**<.0001**
Carbohydrates,*g/d*	227.58 ± 27.53	210.55 ± 28.69	216.31 ± 33.23	**<.0001**
% Energy	50.37 ± 5.94	46.55 ± 6.20	47.66 ± 7.06	**<.0001**
Fat, *g/d*	72.60 ± 10.66	74.87 ± 10.89	74.27 ± 12.72	**0.044**
% Energy	36.79 ± 5.21	37.76 ± 5.65	37.60 ± 6.19	0.082
Protein, *g/d*	65.57 ± 8.49	76.71 ± 11.52	71.76 ± 10.95	**<.0001**
% Energy	14.18 ± 2.10	16.86 ± 2.48	15.80 ± 2.48	**<.0001**
Animal protein, *g/d*	32.50 ± 10.02	46.92 ± 12.88	42.53 ± 13.16	**<.0001**
Plant protein, *g/d*	33.09 ± 5.35	29.81 ± 4.91	29.26 ± 5.54	**<.0001**
**Protein sources**				
From grain, *g/d*	15.76 ± 4.30	15.01 ± 4.56	17.24 ± 4.72	**<.0001**
From beans, *g/d*	4.62 ± 3.82	4.27 ± 3.63	3.80 ± 3.35	**0.009**
From vegetables, *g/d*	5.28 ± 2.68	4.84 ± 2.12	4.20 ± 2.06	**<.0001**
From fruits, *g/d*	2.25 ± 1.12	1.83 ± 1.12	1.50 ± 0.87	**<.0001**
From red meat, *g/d*	9.85 ± 5.27	14.64 ± 7.46	23.06 ± 9.60	**<.0001**
From poultry, *g/d*	2.61 ± 2.04	8.47 ± 4.74	3.43 ± 2.43	**<.0001**
From aquatic products, *g/d*	5.01 ± 3.78	11.60 ± 8.87	4.98 ± 3.57	**<.0001**
From eggs, *g/d*	6.30 ± 3.67	4.28 ± 2.94	4.66 ± 3.03	**<.0001**
From dairy, *g/d*	8.76 ± 5.45	7.96 ± 5.04	6.43 ± 4.64	**<.0001**
From nuts and seeds, *g/d*	4.84 ± 4.88	3.45 ± 4.17	2.17 ± 2.95	**<.0001**

[1] Values were presented as mean± standard deviation.

[2] Energy-adjusted intake estimated by the residual method.

Supplementary Table S4 Dietary consumption of pregnant women by dietary protein patterns[1].

**Food groups**	Overall (*N*=1014)	Plant-dairy-eggs (*N*=302)	White meat (*N* =297)	Red meat (*N* =415)	*P*
Grain, *g/d*	202.0 ± 78.5	198.9 ± 78.7	195.7 ± 77.0	208.8 ± 79.1	0.066
Beans, *g/d*	21.9 ± 23.2	28.3 ± 27.7	23.5 ± 23.3	16.3 ± 17.6	**<.0001**
Vegetables, *g/d*	224.5 ± 128.9	239.5 ± 148.2	234.2 ± 124.2	206.8 ± 114.6	**0.001**
Fruits, *g/d*	269.6 ± 173.0	317.0 ± 180.9	281.8 ± 185.4	226.6 ± 146.0	**<.0001**
Red meat, *g/d*	92.7 ± 61.1	51.7 ± 33.6	83.8 ± 48.2	128.7 ± 63.7	**<.0001**
Poultry, *g/d*	26.0 ± 24.6	13.4 ± 12.0	48.2 ± 29.7	19.4 ± 15.1	**<.0001**
Aquatic products, *g/d*	43.7 ± 41.1	30.5 ± 24.1	72.9 ± 57.1	32.5 ± 22.7	**<.0001**
Eggs, *g/d*	39.0 ± 26.7	47.7 ± 29.9	33.8 ± 23.6	36.3 ± 24.8	**<.0001**
Dairy, *g/d*	212.9 ± 155.8	238.9 ± 163.6	226.6 ± 151.0	184.3 ± 148.9	**<.0001**
Nuts and seeds, *g/d*	12.0 ± 20.3	11.1 ± 20.5	13.6 ± 18.4	11.5 ± 21.4	0.255

[1] Values were presented as mean± standard deviation.

Supplementary Table S5 The odds ratios of GDM across three dietary patterns.

Subgroup	Dietary Patterns			$P_{interaction}$
	Plant protein	White meat protein	Red meat protein	
Pre-pregnancy BMI				0.645
Obese or overweight	1.00	**8.043 (1.487-43.49)**	**8.539 (1.459-49.98)**	
Normal or thin	1.00	1.456 (0.774-2.736)	1.521 (0.851-2.717)	

Participants were categorized into two weight groups according to pre-pregnancy BMI using the Chinese criteria for adults: underweight or normal (<23.9 kg/m^2), overweight or obese (≥24.0 kg/m^2).

Model was adjusted for age, gestational age, pre-pregnancy BMI, GDM in a previous pregnancy, family history of diabetes, physical activities, smoking status, alcohol use during pregnancy, dietary energy intake, protein to energy ratio, fat to energy ratio, carbohydrate to energy ratio, fiber, cholesterol, monthly household income, and educational level.

Supplementary Table S6 The odds ratios of GDM across three dietary patterns when compared to women with red meat protein pattern.

	Dietary Protein Patterns		
Model	Red meat	White meat	Plant-dairy-eggs
GDM (*N*,%)	80 (19.28%)	62 (20.88%)	49 (16.23%)
Unadjusted	1.00	1.11 (0.76-1.60)	0.81 (0.55-1.20)
Adjusted			
Model 1	1.00	1.18 (0.79-1.76)	0.66 (0.43-1.02)
Model 2	1.00	1.15 (0.77-1.71)	**0.63 (0.41-0.98)**
Model 3	1.00	1.07 (0.70-1.64)	**0.55 (0.32-0.92)**
Model 4	1.00	1.02 (0.66-1.58)	**0.56 (0.33-0.95)**

Model 1 was adjusted for age, gestational age, pre-pregnancy BMI;

Model 2 was further adjusted forGDM in a previous pregnancy, family history of diabetes;

Model 3 was further adjusted for physical activities, smoking status, alcohol use during pregnancy, dietary energy intake, protein to energy ratio, fat to energy ratio, carbohydrate to energy ratio, fiber, and cholesterol;

Model 4 was further adjusted for monthly household income, andeducational level.

Supplementary Table S7 The odds ratios of GDM across three dietary patterns when compared to women with white meat protein pattern.

	Dietary Protein Patterns		
Model	White meat	Red meat	Plant-dairy-eggs
GDM (*N*,%)	62 (20.88%)	80 (19.28%)	49 (16.23%)
Unadjusted	1.00	0.91 (0.62-1.31)	0.73 (0.49-1.11)
Adjusted			
Model 1	1.00	0.85 (0.57-1.27)	**0.56 (0.35-0.88)**
Model 2	1.00	0.87 (0.58-1.31)	**0.55 (0.35-0.88)**
Model 3	1.00	0.94 (0.61-1.43)	**0.51 (0.29-0.89)**
Model 4	1.00	0.98 (0.63-1.52)	**0.55 (0.31-0.96)**

Model 1 was adjusted for age, gestational age, pre-pregnancy BMI;

Model 2 was further adjusted forGDM in a previous pregnancy, family history of diabetes;

Model 3 was further adjusted for physical activities, smoking status, alcohol use during pregnancy, dietary energy intake, protein to energy ratio, fat to energy ratio, carbohydrate to energy ratio, fiber, and cholesterol;

Model 4 was further adjusted for monthly household income, andeducational level.
